# A Doctor’s Experience

**Published:** 1890-09

**Authors:** A. K. Bell

**Affiliations:** Madison, Ga.


### Clinical Department.

### A DOCTOR'S EXPERIENCE.

BY A. K. BELL, M. D., MADISON, GA.

I write these few lines to show how sensitive some people's eyes are to the action of sulphate atrophine. Having had an uneasy feeling of the eyes and a free discharge of water from them, I decided to use a week solution of atrophine. A pellet 1-150 of a grain was dissolved in one drachm of water, and one drop of this solution was dropped into each eye. In an hour or two the pupil was so dilated that I was unable to read any medium-size print; could not discern objects plainly in the bright light; was called upon to make a slight operation, but could not see well enough. This condition lasted for forty-eight hours. A hypodermic injection of one-fourth grain of morphia was taken, but with no effect upon the eye. A fourth of grain of morphine was dissolved in one drachm of water and a few drops of the solution was put into each eye, with no effect.

Some time ago, (about five years) I had the same thing to happen to me—the solution was made by an occulist, and for two or three days I was unable to attend to ordinary duty. After keeping in a dark room it gradually wore off.

Hoping that no one else will have my unpleasant experience.

---

Pineapple juice is said to be largely employed by the negroes of Louisiana in cases of diphtheria. The success of this popular treatment is said to be eminently satisfactory.—*Ex.*

---

DEATH DUE TO VESICAL ENDOSCOPY.—Dr. Albarran reports the death of a patient who had submitted to vesical endoscopy. The urine, examined after death, contained pyogenic bacteria.

